# Artificial Intelligence in Gynecology and Obstetrics: Medico-Legal, Ethical, and Governance Challenges—A Narrative Review

**DOI:** 10.3390/healthcare14213760

**Published:** 2026-11-04

**Authors:** Giusi Santangelo, Gaia De Angelis, Ludovico Muzii, Violante Di Donato, Giorgia Perniola, Gabriele Napoletano, Michele Treglia, Nicola Di Fazio, Aniello Maiese, Fabio Del Duca

**Affiliations:** 1 Department of Gynecological, Obstetrical and Urological Sciences, Sapienza University of Rome, Viale Regina Elena 336, 00161 Rome, Italyludovico.muzii@uniroma1.it (L.M.); violante.didonato@uniroma1.it (V.D.D.); giorgia.perniola@uniroma1.it (G.P.); 2 Department of Anatomical, Histological, Forensic and Orthopedic Sciences, Sapienza University of Rome, Viale Regina Elena 336, 00161 Rome, Italy; gaia.deangelis@uniroma1.it (G.D.A.); aniello.maiese@uniroma1.it (A.M.); 3 Department of Biomedicine and Prevention, University of Rome “Tor Vergata”, 00133 Rome, Italy; michelemario@hotmail.it (M.T.); fabio.delduca@uniroma1.it (F.D.D.); 4 Department of Life Sciences, Health and Health Professions, Link Campus University, 00165 Rome, Italy; n.difazio@unilink.it

**Keywords:** artificial intelligence, gynecology, obstetrics, medical liability, informed consent, reproductive health, algorithmic bias, data protection, human oversight, governance

## Abstract

**Background:** Artificial intelligence (AI) is entering gynecologic and obstetric imaging, risk prediction, decision support, robotic surgery, remote monitoring, and patient communication. An estimated 260,000 maternal deaths occurred worldwide in 2023, and in England obstetric claims account for 11% of clinical negligence claims but 55% of their value. AI errors, bias, or opacity in this specialty therefore carry major clinical and legal consequences. **Objective:** To synthesize the principal medico-legal and bioethical challenges associated with AI in gynecology and obstetrics and to propose a lifecycle governance framework for responsible clinical adoption. **Methods:** A narrative review with thematic synthesis was conducted. PubMed, Scopus, and Google Scholar were searched for records published from January 2020 to August 2026, supplemented by citation chaining and official legal databases. Two reviewers screened 131 records against predefined eligibility criteria; 41 sources (36 publications and 5 legal instruments) were included and analyzed thematically. Selection is reported in a PRISMA-style flow diagram. **Results:** Six connected risk domains were derived from five prespecified themes, namely validation and generalizability; professional competence and automation bias; distributed liability and evidentiary opacity; informed consent and patient autonomy; privacy and cybersecurity of reproductive data; and discrimination, access, and reproductive justice. Generative AI adds risks of hallucination, non-reproducibility, persuasive framing, and undocumented use. Existing law does not eliminate clinician responsibility: accountability is shared across developers, institutions, and professionals, but clinical judgment and documented oversight remain central. In the European Union, Medical Device Regulation and GDPR obligations already apply, whereas AI Act requirements for AI-enabled medical devices apply from August 2028 and the revised Product Liability Directive covers products placed on the market after 9 December 2026; causation and professional fault remain governed by national law. **Conclusions:** Safe adoption requires more than technical accuracy. We translate the findings into seven actionable institutional requirements, namely accountable ownership, minimum local validation, role-specific training, auditable documentation, equity monitoring, post-deployment surveillance, and incident reporting. AI should augment—not displace—clinically accountable and relational care.

## 1. Introduction

Artificial intelligence (AI) now encompasses a spectrum of technologies used in health care, including rule-based systems, conventional machine-learning models, deep neural networks, computer vision, natural-language processing, and generative large language models (LLMs). In gynecology and obstetrics, these tools are being developed for ultrasound and magnetic-resonance image interpretation, fetal surveillance, prediction of pre-eclampsia and preterm birth, mode-of-delivery estimation, oncology, triage, robotic surgery, remote monitoring, and patient education [1,2,3,4,5,6,7,8,9]. The relevant policy question is therefore no longer whether clinicians will encounter AI, but under what conditions its use is clinically reliable, ethically defensible, and legally accountable. The specialty creates an unusually demanding test case for responsible AI. Many decisions are time-sensitive and have high consequences; diagnostic error may affect fertility, pregnancy outcomes, cancer prognosis, or access to urgent care. Pregnancy also involves interdependent maternal and fetal risks without erasing the pregnant patient’s autonomy. Reproductive choices are value-laden and legally variable, while menstrual, sexual, fertility, genetic, and abortion-related information can expose patients to stigma, discrimination, surveillance, or prosecution if misused. Finally, obstetrics and gynecology already experience substantial malpractice pressure, making uncertainty about algorithmic responsibility particularly consequential [10,11,12,13,14,15]. In England, obstetric claims represented 11% of clinical negligence claims notified to NHS Resolution in 2025/26 but 55% of their total value [16], and an estimated 260,000 maternal deaths occurred worldwide in 2023 [17]. AI can reduce variability, identify complex patterns, and broaden access to expertise, yet performance in a development dataset is not equivalent to clinical benefit. Dataset shift, missingness, low prevalence, label error, and differences in equipment or workflow can degrade performance after deployment. Automation bias may cause clinicians to accept an incorrect recommendation, while algorithm aversion may lead them to ignore a useful one. LLMs introduce a different failure mode: fluent but false or incomplete output that may be more persuasive than reliable [18,19,20,21,22]. These risks are not independent. A biased model can undermine justice, a poorly explained output can compromise consent, and an undocumented override can weaken both patient safety and legal defensibility [10,23,24,25]. This narrative review integrates clinical, medico-legal, and bioethical literature with current European regulatory developments. Its objectives are to identify the applications of greatest legal and ethical sensitivity, clarify how responsibility is distributed among clinicians, institutions, and technology providers, and translate broad principles into a practical governance framework for gynecologic and obstetric care. The literature underpinning these objectives was identified through a structured search of three bibliographic databases using predefined keywords and eligibility criteria, as detailed in Section 2.

## 2. Materials and Methods

### 2.1. Review Design

A narrative review design was chosen because the evidence is heterogeneous and includes diagnostic and prognostic studies, systematic and scoping reviews, qualitative research, clinical opinions, ethical analyses, legal scholarship, and binding regulatory instruments. Such sources answer different questions and cannot be meaningfully pooled into a single quantitative estimate. The review was planned and reported with reference to the six items of the Scale for the Assessment of Narrative Review Articles (SANRA) [26], and source selection is reported with an adapted PRISMA 2020 flow diagram.

### 2.2. Search Strategy and Time Frame

Several databases (PubMed, Scopus, and Google Scholar) were searched for records published between 1 January 2020 and 31 August 2026. Search strings combined three concept blocks with Boolean operators, covering (i) artificial intelligence, machine learning, deep learning, large language models, or chatbots; (ii) gynecology, obstetrics, pregnancy, reproductive health, fertility, or women’s health; and (iii) liability, accountability, malpractice, informed consent, privacy, data protection, ethics, bias, equity, or governance. The search string used in PubMed was (“Artificial Intelligence”[Mesh] OR “Machine Learning”[Mesh] OR “artificial intelligence” OR “machine learning” OR “deep learning” OR “large language model*” OR chatbot* OR ChatGPT) AND (gynecolog* OR obstetric* OR pregnan* OR “reproductive health” OR fertility OR “women’s health”) AND (liabilit* OR accountab* OR malpractice OR “informed consent” OR privacy OR “data protection” OR ethic* OR bias OR equit* OR governance).

The lower limit of 2020 was chosen because it marks both the transition of AI in obstetrics and gynecology from proof-of-concept studies to clinical evaluation and the start of the current EU regulatory cycle. This cycle includes the European Commission White Paper on AI (February 2020), the AI Act proposal (April 2021), the date of application of the Medical Device Regulation (May 2021), and the public release of general-purpose large language models (late 2022). Earlier foundational publications and legal instruments (e.g., the GDPR and Italian Laws No. 24/2017 and No. 219/2017) were retained when they remained the applicable legal basis or were needed to frame key concepts.

### 2.3. Eligibility Criteria and Source Selection

Inclusion criteria were (i) peer-reviewed articles in English addressing AI in gynecology, obstetrics, or reproductive and women’s health, or addressing medico-legal, ethical, or regulatory mechanisms directly transferable to this specialty; (ii) empirical studies (diagnostic, prognostic, evaluation, survey, or qualitative), systematic, scoping, or narrative reviews, and legal or ethical analyses; and (iii) binding EU or Italian legal instruments on medical devices, AI, product liability, data protection, professional liability, or informed consent. Exclusion criteria were technical model-development reports without clinical, legal, or ethical analysis; conference abstracts, editorials, and letters without substantive analysis; non-English publications; and records superseded by a more recent or more comprehensive source.

Priority among eligible sources was assigned a priori according to two criteria, namely specialty relevance (specialty-specific sources were preferred over general health-AI sources) and level of evidence, following the conventional hierarchy of systematic and scoping reviews, primary empirical studies, and narrative reviews or expert opinion. For legal questions, binding legal texts were preferred over secondary commentary. Expert opinions and liability analyses were retained because, for many medico-legal and ethical questions, empirical evidence does not yet exist.

**Figure 1:**
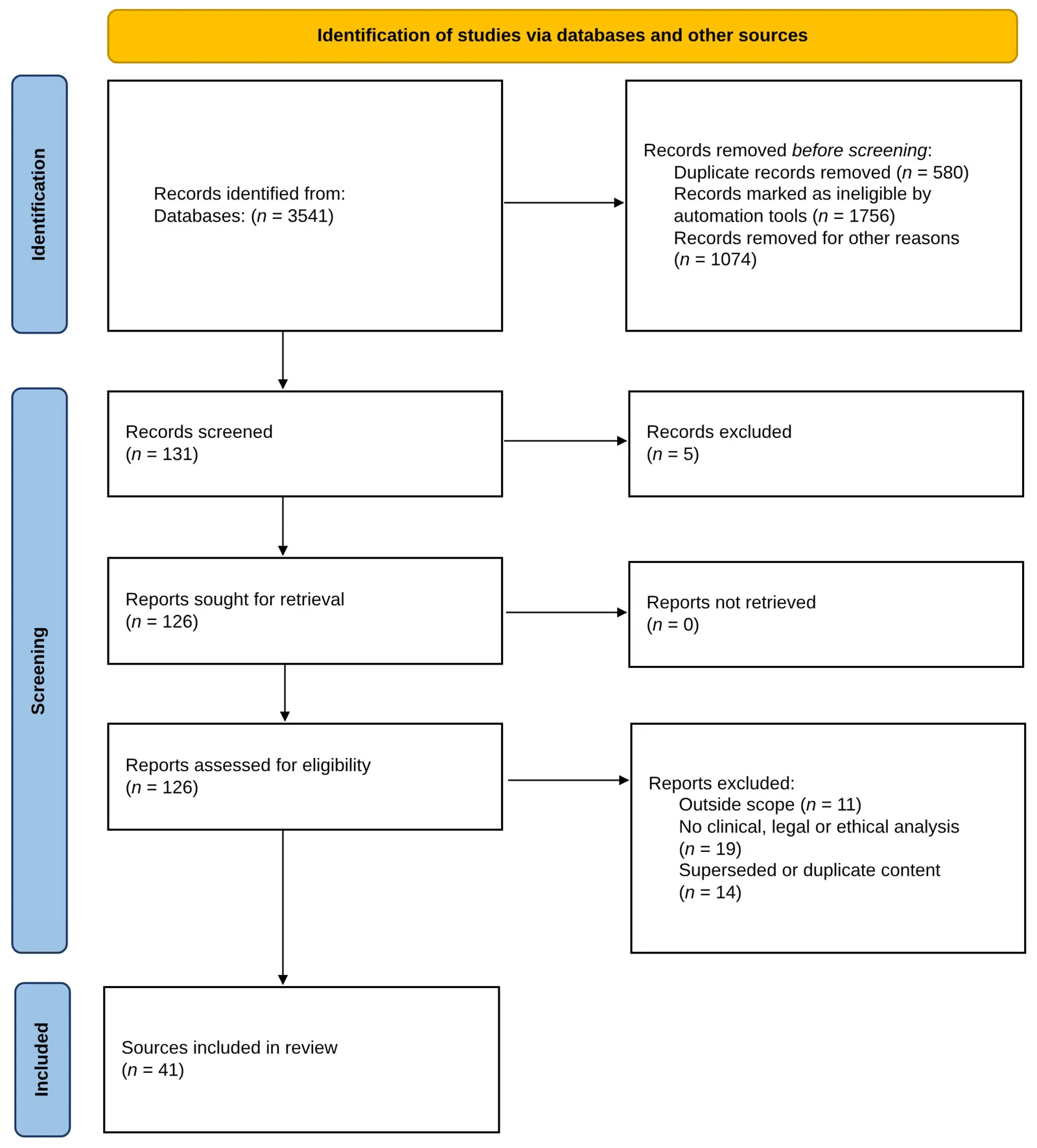
Flow diagram of source identification, screening, and inclusion, adapted from the PRISMA 2020 template. Records identified from each database are reported separately.

Titles and abstracts were screened independently by two authors (G.S. and G.D.A.), followed by full-text assessment of potentially eligible reports. Disagreements were resolved by discussion and, when necessary, by a third author (F.D.D.). Of the 131 records screened, 41 sources were included (Figure 1). These comprised 9 empirical studies, 4 systematic or scoping reviews, 13 narrative, critical, or bibliometric reviews, 10 legal, ethical, or policy analyses and expert opinions, and 5 legal instruments.

### 2.4. Data Charting and Thematic Synthesis

For each included source, the source type, clinical setting, AI application, and medico-legal, ethical, or regulatory issues addressed were charted. Sources were first mapped deductively to five prespecified themes, namely clinical applications, professional accountability and liability, autonomy and informed consent, privacy and data governance, and bias, equity, and trust. Within each theme, issues were then coded inductively and grouped by mechanism of harm. This step produced six risk domains. The theme of clinical applications split into two distinct mechanisms. The first was technical failure of the model, which became the domain of validation and generalizability. The second was failure of the human–machine interaction, which became the domain of professional competence and automation bias. Each of the other four themes matched one domain. Professional accountability and liability became distributed liability and evidentiary opacity. Autonomy and informed consent became informed consent and patient autonomy. Privacy and data governance became privacy and cybersecurity of reproductive data. Bias, equity, and trust became discrimination, access, and reproductive justice. The domains were discussed by all authors until consensus was reached. No meta-analysis or formal risk-of-bias scoring was performed, and the number of citations should not be understood as a measure of evidentiary strength. Recent 2026 publications were included when bibliographic metadata could be verified. The analysis distinguishes empirical findings from clinical opinions and normative proposals and avoids treating technical feasibility as proof of improved patient outcomes.

### 2.5. Legal and Bioethical Analysis

For each EU and Italian instrument, we identified the relevant provisions, their scope, and their date of application, distinguishing obligations already in force (as of October 2026) from forthcoming requirements and from questions that remain open to interpretation. The bioethical analysis applied the four principles of biomedical ethics—autonomy, beneficence, non-maleficence, and justice [27]—complemented by a reproductive-justice lens, to each of the six risk domains; for each principle, we recorded the main tension generated by AI use, the domains involved, and the corresponding safeguard.

## 3. Clinical AI Applications, Risk Mechanisms, and Minimum Safeguards

The medico-legal significance of an AI system depends not only on its reported accuracy but also on its intended purpose, level of autonomy, clinical context, and position in the care pathway. A model that prioritizes a worklist has a different risk profile from one that triggers an emergency intervention or frames counseling about pregnancy termination. The same technology can also shift categories when integrated into a different workflow. Table 1 links major applications to their principal failure modes and minimum human controls.

### 3.1. Imaging, Screening, and Gynecologic Oncology

Computer-vision systems are being studied for fetal biometry, placental location, gynecologic tumor characterization, cervical cytology, mammography, and operative planning. In gynecologic oncology, machine learning may support lesion detection, pathology classification, prognosis, treatment selection, and longitudinal surveillance [28,29]. These applications can improve reproducibility, particularly where specialist expertise is scarce, but they are vulnerable to spectrum bias, differences in scanner vendors and acquisition protocols, and labels that encode historical diagnostic practices. A model trained in high-quality tertiary-center images may fail when deployed in community care or low-resource settings. False reassurance can delay diagnosis; excessive false positives can expose patients to anxiety, invasive procedures, or overtreatment. Imaging models may also perform differently across age, body habits, race or ethnicity, pregnancy stage, and disease prevalence. Equity therefore cannot be inferred from aggregate accuracy. Subgroup calibration, external validation, and prospective assessment of downstream consequences are required [30,31,32,33,34]. Where the model influences diagnosis or treatment, clinicians need access to the original images and sufficient information to challenge the output rather than receiving an unexplained binary label [35].

### 3.2. Obstetric Prediction and Intrapartum Decision Support

Prediction models have been proposed for pre-eclampsia, fetal growth restriction, preterm birth, postpartum hemorrhage, operative delivery, and other adverse outcomes [36,37,38]. The attraction is clear: earlier identification could enable surveillance, prophylaxis, transfer, or timely escalation. However, many models remain retrospective, single-center, or poorly calibrated outside the development population. A high area under the receiver-operating-characteristic curve does not establish that a threshold improves maternal or neonatal outcomes, nor does it show that the intervention triggered by the prediction is beneficial. Cardiotocography and labor-management tools concentrate risk because outputs are generated in real time and can influence urgent operative decisions. Over-alerting can contribute to unnecessary cesarean delivery, while under-alerting can delay intervention. When clinicians and midwives disagree with an algorithm, professional hierarchy and interface design may determine whose judgment prevails. Safe systems should display uncertainty, preserve access to the underlying tracing and clinical context, record recommendations and overrides, and be evaluated for both missed deterioration and intervention burden. Human oversight must be operational: a nominal ability to override is insufficient if staffing, time pressure, or interface design makes critical review unrealistic.

### 3.3. Reproductive Medicine, Remote Monitoring, and FemTech

Fertility prediction, cycle tracking, remote pregnancy monitoring, and consumer FemTech collect large volumes of longitudinal and intimate data. These tools may facilitate self-management and access, but their clinical status is often ambiguous: a wellness application may shape health decisions without being regulated as a medical device. Proprietary risk scores may be difficult for patients and clinicians to interpret, and commercial incentives may favor engagement or data monetization rather than clinical benefit. The risk extends beyond confidentiality. Incomplete datasets may exclude irregular cycles, gender-diverse users, people with disabilities, rural communities, and groups with limited digital access. Reproductive-health tools can therefore reproduce a narrow model of the ‘typical’ user and shift responsibility onto patients who cannot or do not wish to engage continuously with digital monitoring. Equity-oriented design requires participatory development, affordable access, language and disability accommodation, data minimization, and evaluation of who is left out [32,34,38,39].

### 3.4. Generative AI, Chatbots, and Patient Communication

Patients already use general-purpose chatbots to ask about contraception, infertility, pregnancy symptoms, fetal anomalies, abortion, urogynecologic surgery, and mental health. Studies in urogynecology, post-hysterectomy counseling, perinatal depression, and medical abortion show why accessibility and conversational fluency are attractive, but they also highlight variable completeness, readability, consistency, and alignment with expert advice [18,19,20,21,22,23]. LLM output may change with prompt wording or model updates, making reproducibility difficult. In reproductive counseling, apparently neutral language can frame options, omit relevant alternatives, or subtly direct a patient toward a value-laden choice. The problem is not limited to hallucinated facts; an accurate but unbalanced response can still compromise autonomy. General-purpose chatbots should not be treated as autonomous clinical counselors. When institutions deploy them, content boundaries, escalation triggers, source grounding, version control, language testing, and human review are essential. Patients must know when they are interacting with a machine and how to reach a qualified professional [10,21,22].

### 3.5. Robotic and AI-Augmented Surgery

Robotic surgery illustrates how responsibility becomes distributed across the surgeon, team, institution, manufacturer, maintenance chain, and software. Current platforms remain clinician controlled, but AI may increasingly assist with navigation, recognition, risk alerts, or semi-autonomous subtasks. Adverse-event reconstruction requires technical logs, device maintenance records, training documentation, video or telemetry when lawfully retained, and a clear account of human actions. Without interoperable audit trails, causation may be practically impossible to reconstruct [14].

## 4. Medico-Legal Challenges

A thematic synthesis identified six interconnected risk domains. Table 2 links each domain to a representative obstetric or gynecologic scenario, the stakeholders affected, the potential harm, and the corresponding mitigation measures. Validation and generalizability are discussed with the clinical applications in Section 3; the remaining domains are analyzed below.

### 4.1. Standard of Care and Clinical AI Competence

In most legal systems, use of AI does not displace the clinician’s duty to exercise independent professional judgment. A recommendation is evidence to be interpreted, not an actor capable of assuming the clinician’s duty of care. At the same time, the standard of care is dynamic. Once a validated tool becomes widely accepted and incorporated into guidelines, unreasonable failure to use it could potentially be criticized; premature adoption of an inadequately validated tool could also be negligent. The legal question is therefore contextual: what would a reasonably competent practitioner have done with the information, limitations, and resources available at the time? [11,12,24]. Clinical AI competence should include the ability to identify the system’s intended use, recognize out-of-distribution cases, understand the meaning and limitations of performance metrics, interpret uncertainty, detect implausible outputs, communicate the role of AI, protect data, document reliance or override, and report incidents [24,25]. This is not a demand that every gynecologist become a data scientist. It is analogous to the competence required to use ultrasound, fetal monitoring, or a surgical device safely. Institutions share responsibility for providing training, credentialing, and technical support rather than shifting the entire burden to individual clinicians.

### 4.2. Distributed Liability

An AI-related injury may arise from a defective product, poor training data, flawed local integration, inadequate procurement, cybersecurity failure, negligent interpretation, or a combination of factors. Potential defendants can therefore include the clinician, healthcare organization, software developer, medical-device manufacturer, data processor, integrator, or maintenance provider [11,13,14]. Allocation depends on jurisdiction, contractual arrangements, product status, and whether the error was foreseeable and preventable. Three recurring scenarios help clarify responsibility. First, if a clinician follows an obviously implausible output without adequate review, professional negligence may predominate. Second, if a system behaves outside its documented specifications or fails despite appropriate use, product or provider responsibility becomes more salient. Third, if local deployment lacks validation, training, staffing, or monitoring, institutional responsibility may be central. In practice, these categories overlap. Contracts should therefore allocate duties for updates, cybersecurity, incident notification, access to logs, and post-market surveillance, but contractual allocation cannot erase duties imposed by law or professional ethics. In Italy, Law No. 24/2017 structures healthcare liability around patient safety, organizational responsibility, professional conduct, and adherence to guidelines and good clinical practice. AI should be treated as part of the clinical organization and risk-management system, not as a private tool used outside institutional governance. Law No. 219/2017 further reinforces the centrality of informed consent and communication. These national rules interact with EU product, device, AI, and data-protection law rather than being replaced by them [40,41].

EU Member States apply different causation standards and evidentiary presumptions, so the same AI-related adverse event may receive different legal treatment across the EU; the revised Product Liability Directive harmonizes only the producer’s strict liability, not professional or institutional fault.

### 4.3. Explainability, Causation, and Evidentiary Reconstruction

Model opacity affects both care and litigation. Clinicians may not need a complete mathematical explanation, but they need clinically meaningful information: intended purpose, relevant inputs, confidence or uncertainty, known limitations, and the factors that should trigger skepticism. Post hoc visual explanations can be misleading if they create an appearance of transparency without faithfully representing the model’s reasoning. After an adverse event, parties may need to know which model version was active, what data were entered, whether data were missing or transformed, the output and confidence score, alerts displayed, user actions, overrides, software updates, and relevant system performance at that time. Logs must be sufficiently detailed, time synchronized, secure, and retained for a defensible period. Documentation should not become indiscriminate surveillance of clinicians; governance must balance accountability with proportionality, workforce trust, and data minimization. The absence of appropriate records can impede proof of defect, negligence, and causation [12,13,14].

### 4.4. Informed Consent and Disclosure

Not every background algorithm requires a separate signed consent form. Disclosure should be proportionate to materiality. Patients should generally be informed when AI meaningfully influences diagnosis, prognosis, treatment selection, or counseling; when a chatbot or automated system interacts directly with them; when data will be used beyond ordinary care; or when the technology introduces uncertainties a reasonable patient would consider important. Relevant information includes the purpose of the system, its role relative to the clinician, important limitations and alternatives, the possibility of error, human oversight, and how data are handled [10,24,25]. Consent is a process rather than a liability waiver. Dense technical language does not create understanding, and AI-generated consent documents are not reliable unless professionally reviewed. In fertility, prenatal diagnosis, and pregnancy termination, the clinician must preserve space for values, ambivalence, and questions. A patient’s refusal of an AI-mediated pathway should be respected when a clinically reasonable alternative exists, without creating unjustified delay or inferior care.

First, disclosure is legally required when the patient interacts directly with an AI system (Article 50(1) of the AI Act, applicable since 2 August 2026) and, in Italy, whenever AI is used in the patient’s care (Article 7 of Law No. 132/2025). Second, it is ethically required when an AI output materially shapes a diagnosis, risk estimate, or treatment option discussed with the patient. Third, it is advisable when data are reused beyond direct care. Meaningful decision-making authority is preserved when the patient receives the AI-informed estimate together with its uncertainty and the clinician’s own assessment, can request a human-only assessment, and is not penalized for declining an AI-mediated pathway. When an AI recommendation conflicts with clinical judgment, the clinician should re-examine the inputs and the patient, seek a second human opinion where time allows, follow the clinically justified course, and document the divergence and its rationale; outputs should be neither silently discarded nor silently followed. When the recommendation, the clinician’s judgment, and the patient’s preferences diverge, the informed choice of a patient with decision-making capacity prevails within the range of clinically reasonable options, and the discussion should be documented.

### 4.5. Data Protection, Cybersecurity, and Secondary Use

Reproductive and genetic data are particularly sensitive because harm can extend beyond the individual to partners, relatives, and communities. The General Data Protection Regulation (GDPR) treats health and genetic data as special categories and requires a lawful basis, purpose limitation, data minimization, security, and respect for data-subject rights. High-risk processing may require a data-protection impact assessment. Article 22 protections concerning solely automated decisions require careful analysis, although meaningful clinician involvement may mean a clinical decision-support process is not solely automated [42]. De-identification does not eliminate risk when datasets are rich, longitudinal, or linkable. Federated learning, synthetic data, and privacy-preserving computation can reduce some exposures but introduce new validation and governance questions. Cybersecurity is also a patient-safety issue: manipulated inputs, model extraction, prompt injection, ransomware, or compromised updates can alter recommendations. Procurement should address data location, subcontractors, retention, reuse for model training, breach response, and the institution’s ability to delete or retrieve data at the end of the contract.

### 4.6. Bias, Discrimination, and Access

Bias can enter through sampling, labels, measurement, missingness, outcome definitions, and deployment. Historical data may encode unequal access, undertreatment, or differential diagnosis. A model can therefore be statistically accurate overall while producing systematically worse errors for underserved groups. In obstetric triage or resource allocation, these errors may compound existing disparities in maternal morbidity and mortality [30,35]. Fairness is not solved by removing race, ethnicity, sex, or socioeconomic variables. Proxy features may remain, and protected variables can be necessary to identify unequal performance. Evaluation should report subgroup sensitivity, specificity, calibration, false-positive and false-negative burdens, and access effects. Institutions should predefine acceptable disparities, investigate their causes, and suspend or constrain a system when harm exceeds thresholds. Equity auditing must include intersectional groups and patients not represented in digital datasets, not only the largest demographic categories.

## 5. Bioethical Analysis

Following the approach described in Section 2.5, each risk domain was examined against the four principles of biomedical ethics and a reproductive-justice lens, and the main observations are summarized in Table 3. The classical principles of autonomy, beneficence, non-maleficence, and justice remain useful, but AI reveals tensions between them. A highly accurate system may advance beneficence while compromising autonomy if its role is hidden. A tool may reduce average harm yet worsen outcomes for a minority group. Transparency may support trust but can conflict with intellectual property claims or overwhelm patients with technical details. Ethical analysis must therefore address the entire sociotechnical system rather than the model alone. Reproductive decisions frequently concern identity, family, disability, religion, and future life plans. Prediction can be experienced as recommendation, particularly when delivered by an authoritative interface. Systems should therefore support deliberation rather than optimize a single outcome. Counseling tools must present reasonable options, disclose uncertainty, avoid manipulative personalization, and enable a patient to pause or seek human support. For pregnancy, concern for fetal outcome does not justify obscuring the pregnant patient’s rights, values, or decisional authority. Human review is essential but not infallible. It is unrealistic to make clinicians the sole safety barrier for systems that operate at high volume, change silently, or present outputs in ways that encourage acceptance. Beneficence requires evidence that the AI-enabled pathway improves meaningful outcomes, not merely technical performance. Non-maleficence requires fail-safe design, workload assessment, clear escalation, and monitoring for delayed or indirect harms such as anxiety, overdiagnosis, deskilling, alert fatigue, and unnecessary intervention. A justice analysis asks whether benefits and burdens are distributed fairly; a reproductive-justice lens adds whether people have the real ability to have children, not have children, and parents in safe and supportive environments. Digital exclusion, criminal-legal surveillance, restrictive abortion environments, and commercialization of fertility data can make the same tool empowering for one group and dangerous for another [34,35,39]. Responsible innovation should assess affordability, infrastructure, language, disability access, legal context, and the consequences of non-use. Trust depends on competence, honesty, and relational presence. AI can create time for conversation if it reduces administrative burden, but it can also insert a third party whose incentives and data practices are opaque. Clinicians should be able to explain how a system affected care and accept responsibility for the final clinical plan. Institutions should avoid presenting automation as neutral or inevitable. The goal is not maximal automation; it is a care relationship in which technology strengthens safety without displacing empathy, contextual judgment, or shared decision-making.

## 6. Regulatory Landscape

In the European Union, AI in clinical care is governed through overlapping regimes. Software with an intended medical purpose may be a medical device under Regulation (EU) 2017/745 and must satisfy classification, conformity assessment, clinical evaluation, quality-management, vigilance, and post-market obligations [40,43]. Under Rule 11 of Annex VIII, software that provides information used for diagnostic or therapeutic decisions is at least class IIa, and class IIb or III when such decisions may cause serious or irreversible deterioration of health; manufacturers must also meet clinical-evaluation (Article 61), post-market surveillance (Articles 83–86), and vigilance (Article 87) obligations, and a health institution that manufactures or modifies software for in-house use must meet the conditions of Article 5(5). The AI Act, Regulation (EU) 2024/1689, adds a risk-based framework. Under Article 6(1) and Annex I, AI systems that are, or are safety components of, medical devices requiring notified-body conformity assessment—in practice, software of class IIa or higher—are classified as high-risk [44]. Requirements include risk management, data governance, technical documentation, logging, transparency to deployers, human oversight, accuracy, robustness, cybersecurity, and post-market monitoring [45]. These requirements are set out in Articles 9–15 and 72; deployers, including hospitals, must use high-risk systems according to the instructions for use, assign human oversight to competent staff, monitor operation, and keep automatically generated logs for at least six months (Article 26). The obligations are staggered. Prohibited practices and AI-literacy duties (Articles 4–5) have applied since 2 February 2025 and transparency duties for systems interacting with patients (Article 50) since 2 August 2026, whereas, following the Digital Omnibus on AI, high-risk requirements apply from 2 December 2027 to Annex III systems, such as emergency patient-triage systems, and from 2 August 2028 to AI-enabled medical devices [46]. Until then, the conformity of AI-enabled medical devices is assessed under the MDR alone. The revised Product Liability Directive (EU) 2024/2853 expressly brings software, including AI, within the product-liability framework and addresses evidentiary difficulties through disclosure and presumptions in defined circumstances [47]. The Directive must be transposed by 9 December 2026 and applies to products placed on the market after that date; it qualifies software as a product (Article 4), enables courts to order disclosure of evidence (Article 9), and presumes defectiveness or causation where the claimant faces excessive difficulties owing to technical or scientific complexity (Article 10(4)). It complements rather than replaces professional negligence and institutional liability. The proposed AI Liability Directive was formally withdrawn by the Commission in October 2025 [48]; consequently, causation and fault remain substantially dependent on national law. The GDPR governs personal-data processing, including the conditions for processing health and genetic data (Article 9), safeguards concerning solely automated decisions (Article 22), and data-protection impact assessments (Article 35), while sectoral cybersecurity, research, employment, and consumer rules may also apply [42]. From 2029, the European Health Data Space Regulation will add a framework for the secondary use of electronic health data, including for training AI systems [49]. Regulatory compliance is necessary but not sufficient for ethical deployment. A device can bear a conformity mark yet perform poorly in a local population or workflow. Conversely, local customization can change intended use or create new regulatory responsibilities. Procurement, validation, and change control must therefore involve legal, clinical, technical, data-protection, and quality expertise.

## 7. A Lifecycle Governance Framework

Governance should begin before purchase and continue until decommissioning. A multidisciplinary AI committee should define acceptable uses, classify clinical risk, review evidence, and assign accountable owners. High-risk obstetric and gynecologic applications require local validation on representative data, workflow simulation, usability testing, subgroup analysis, and a predefined rollback plan. Validation should evaluate the entire pathway—including clinician response and patient outcomes—not only the model in isolation [25,50]. Deployment should be gradual, with trained users, clear scope, escalation procedures, and explicit rules for overrides. Documentation should capture the system and version, clinically relevant input and output, the clinician’s interpretation when the output materially affects care, and deviations that are important to patient safety. The aim is reconstructability, not indiscriminate defensive documentation. Post-deployment surveillance should monitor technical performance, calibration, subgroup disparities, alert burden, overrides, near misses, adverse events, and unexpected workflow effects. Material model updates require renewed validation. Patients and frontline staff need accessible reporting routes, and serious incidents must be escalated to manufacturers and authorities as required. Table 4 summarizes role-specific responsibilities.

To make the framework actionable, Table 5 translates it into seven minimum institutional requirements, each with an accountable owner and a verifiable indicator. The requirements are designed to be compatible with the deployer obligations of the AI Act, the post-market and vigilance duties of the MDR, and the clinical risk-management function required in Italy by Law No. 24/2017.

## 8. Discussion

The literature shows a persistent gap between technical development and evidence of improved outcomes. Obstetric and gynecologic AI research is expanding rapidly, yet many studies remain retrospective or evaluate performance under conditions that do not reflect routine care [4,8,12,33]. The most mature ethical and legal arguments therefore concern foreseeable mechanisms of harm rather than large bodies of adjudicated cases. This is not a reason to delay all use; it is a reason to adopt proportionate, evidence-sensitive governance. Responsibility should be shared but not diluted. Developers are best placed to manage training-data quality, software design, documentation, and update safety. Institutions control procurement, integration, staffing, access, local validation, and monitoring. Clinicians control contextual interpretation, communication, and the final care plan. Patients contribute values and preferences but should not be made responsible for detecting system defects. Regulators and professional bodies set minimum requirements and standards. When each actor assumes that another will catch the error, the result is an accountability gap. The concept of meaningful human control offers a practical bridge between law and ethics. Control is meaningful when the user has sufficient competence, time, information, authority, and an effective alternative. A clinician cannot meaningfully override a system if the underlying evidence is inaccessible, if the interface conceals uncertainty, if staffing makes review impossible, or if organizational culture punishes deviations. Human oversight must therefore be designed and resourced, not merely declared. Generative AI deserves specific governance because it is general purpose, conversational, and prone to confident error. Institutional use should favor constrained tasks, retrieval from approved sources, output labeling, auditability, privacy-protective configurations, and mandatory professional review. Public-facing tools require clear limitations and urgent-care escalation. They should never be the sole pathway for decisions involving emergency symptoms, fetal anomaly counseling, pregnancy termination, cancer treatment, or other high-stakes care. Three generative-AI risks deserve specific attention. Hallucination—fluent but fabricated content—is particularly dangerous in patient-facing counseling and in AI-drafted clinical documentation, where errors propagate through the medical record. Automation bias is not confined to inexperienced users. In a reader study of AI-supported mammography, accuracy fell from about 80% to below 20% among inexperienced radiologists, and to 45.5% among very experienced radiologists, when the system suggested an incorrect category [51]. Finally, inadequate documentation—undisclosed use of general-purpose chatbots, unsaved prompts, or unlabeled AI-generated text—undermines both clinical continuity and evidentiary reconstruction; institutions should therefore require labeling of AI-generated content, retention of prompts and outputs for clinically relevant uses, and explicit clinician sign-off. Future research should move beyond model accuracy toward prospective clinical effectiveness, patient-reported outcomes, equity, workload, and cost. Studies should compare AI-enabled pathways with current care, evaluate subgroup calibration and error burdens, and report how clinicians actually use or override recommendations. Longitudinal surveillance is needed because performance can drift as populations, equipment, clinical practice, and software change. Legal and ethical research should examine real incident reports and claims, standards for disclosure, patient preferences, documentation burden, and the evidentiary value of audit logs. Comparative analyses should address how AI Act, medical-device, product-liability, GDPR, and national malpractice regimes interact. Qualitative work should include patients facing infertility, pregnancy complications, gynecologic cancer, disability-related prenatal counseling, and abortion care, as well as midwives, nurses, trainees, and clinicians in resource-constrained settings. Finally, professional organizations should develop specialty-specific benchmarks for clinical AI competence and minimum reporting standards. Independent test datasets, incident registries, and transparent model cards could reduce duplicated evaluation and make failures visible across institutions. Public and patient participation should be embedded in priority setting, procurement, and oversight.

This review has limitations. First, as a narrative review it is susceptible to selection bias. Although the search, eligibility criteria, and selection process are reported, relevance ranking in Google Scholar and citation chaining may favor highly cited or recent sources, and non-English legal and ethical literature was excluded. The legal analysis reflects the law as of October 2026 and focuses on the EU; national transposition of the Product Liability Directive and the staggered application of the AI Act may change this picture, and the findings may not transfer to other jurisdictions. Published evidence on AI-related litigation in gynecology and obstetrics is scarce, so the liability scenarios rely on legal reasoning rather than on adjudicated cases.

## 9. Conclusions

This review screened 131 records and synthesized 41 sources, including 9 empirical studies, 4 systematic or scoping reviews, and 5 legal instruments. Qualitatively, six interconnected risk domains emerged—validation and generalizability; professional competence and automation bias; distributed liability and evidentiary opacity; informed consent and patient autonomy; privacy and cybersecurity of reproductive data; and discrimination, access, and reproductive justice—with generative AI acting as a cross-cutting amplifier. Legally, the clinician remains responsible for the final decision, and institutions bear growing organizational duties. Causation and fault remain governed by national law. Practically, the findings translate into seven minimum institutional requirements, namely accountable ownership, local validation, role-specific training, auditable documentation, equity monitoring, post-deployment surveillance, and incident reporting. Under these conditions, AI can augment professional care while preserving patient autonomy, safety, justice, and trust.

## Figures and Tables

**Table 1:** AI applications across gynecologic and obstetric domains.

Clinical Domain	Potential Value	Principal Medico-Legal and Ethical Risks	Minimum Safeguards
Imaging and oncology	Detection, classification, prognosis, workflow prioritization	Spectrum and device bias; missed or false-positive diagnoses; opaque outputs; delayed care	External and local validation; subgroup analysis; access to source images; calibrated uncertainty; prospective outcome monitoring
Obstetric prediction and CTG	Earlier risk identification; decision support in time-critical care	Automation bias; alert fatigue; unnecessary intervention; delayed escalation; maternal–fetal trade-offs	Real-time human review; display of uncertainty; escalation rules; logging of alerts and overrides; workload and intervention-burden monitoring
Fertility, FemTech, and remote monitoring	Access, self-management, longitudinal monitoring	Commercial use of reproductive data; exclusion; unclear device status; digital divide; surveillance	Data minimization; explicit secondary-use rules; accessibility testing; participatory design; clinically safe non-digital alternatives
LLMs and chatbots	Education, navigation, plain-language support	Hallucination; inconsistency; persuasive framing; privacy leakage; delayed emergency care	Disclosure of machine interaction; approved knowledge sources; version control; human review; urgent-care escalation; prohibition on autonomous high-stakes counseling
Robotic and AI-augmented surgery	Precision, navigation, intraoperative support	Shared-control failures; training deficits; device/software defects; inadequate event reconstruction	Credentialing; simulation; maintenance and update control; interoperable technical and clinical logs; incident review

CTG, cardiotocography; LLM, large language model.

**Table 2:** Risk domains linked to representative clinical scenarios, affected stakeholders, potential harms, and mitigation measures.

Risk Domain	Representative Clinical Scenario	Stakeholders Affected	Potential Harm	Mitigation Measures
1. Validation and generalizability	Fetal-biometry or adnexal-mass imaging model trained on tertiary-center images is deployed in a community hospital with different ultrasound equipment	Patients; sonographers; gynecologists; institution	Missed fetal growth restriction or malignancy; false reassurance; unnecessary surgery	External and local validation on representative data; subgroup calibration; access to source images; rollback plan
2. Professional competence and automation bias	AI-assisted CTG interpretation during labor labels a trace as reassuring and the team accepts it despite clinical signs of fetal compromise	Pregnant patient; fetus and neonate; midwives; obstetricians	Delayed operative delivery and hypoxic injury; or unnecessary cesarean delivery after over-alerting	Role-specific training; display of uncertainty; escalation rules; protected time for review; logging of overrides
3. Distributed liability and evidentiary opacity	A pre-eclampsia risk-prediction tool is silently updated by the vendor; a missed high-risk classification leads to omission of aspirin prophylaxis	Patient; clinician; institution; manufacturer	Preventable maternal morbidity; impossibility of reconstructing which model version produced the output; contested allocation of liability	Version control and change notification in contracts; time-synchronized audit logs; retention policy; joint incident review with the vendor
4. Informed consent and patient autonomy	An AI risk score or chatbot is used in prenatal-screening or pregnancy-termination counseling without disclosure	Pregnant patient; partner; counselor	Undisclosed influence on value-laden decisions; framing effects; invalid consent	Proportionate disclosure of the AI role; balanced presentation of options; human counselor; documentation of preferences; right to a non-AI pathway
5. Privacy and cybersecurity of reproductive data	Fertility or cycle-tracking data are reused for model training or accessed by third parties; ransomware disables a perinatal monitoring system	Patients; partners and relatives; institution; app provider	Disclosure of reproductive or abortion-related information; stigma; legal exposure; interruption of monitoring	DPIA; data minimization; secondary-use restrictions; encryption and access control; breach-response and downtime procedures
6. Discrimination, access, and reproductive justice	An obstetric triage or postpartum-hemorrhage model under-performs in minority, migrant, or low-literacy patients; care pathway available only digitally	Underserved groups; institution; regulators	Systematically higher false-negative rates; wider disparities in maternal morbidity; exclusion from care	Subgroup performance reporting; predefined disparity thresholds; equity audits; accessible non-digital alternatives; participatory design
Cross-cutting risk (generative AI)	An ambient AI scribe or LLM drafts a postpartum discharge summary that omits a hypertensive reading or includes a hallucinated medication	Patient; clinician; institution	Missed follow-up of postpartum pre-eclampsia; inaccurate medical record with evidentiary value	Mandatory clinician review and sign-off; labeling of AI-generated text; retention of drafts and prompts; approved tools only

CTG, cardiotocography; DPIA, data protection impact assessment; LLM, large language model.

**Table 3:** Bioethical analysis with the main tensions observed, risk domains involved, and corresponding safeguards.

Principle	Main Tension Observed	Safeguards
Autonomy	Hidden or persuasive AI framing can steer value-laden reproductive choices; prediction may be experienced as recommendation	Proportionate disclosure; balanced presentation of options; right to a human-only pathway; shared decision-making
Beneficence	Technical accuracy is used as a proxy for patient benefit; few prospective outcome studies	Evidence that the AI-enabled pathway improves meaningful maternal, neonatal, or oncological outcomes before routine use
Non-maleficence	Indirect harms such as over-alerting, unnecessary intervention, deskilling, anxiety, and data breaches	Fail-safe design; workload assessment; escalation rules; monitoring of delayed and indirect harms; cybersecurity
Justice and reproductive justice	Unequal performance and access; commercialization of fertility data; surveillance in restrictive legal environments	Subgroup audits; affordable and accessible alternatives; data minimization; assessment of the legal context
Trust and accountability (cross-cutting)	Opaque developer incentives; diffusion of responsibility among actors	Clear role of AI in care; named accountable owner; transparent incident handling

**Table 4:** Responsibility and documentation across the AI lifecycle.

Actor	Core responsibilities	Key Evidence to Retain
Developer/manufacturer	Intended use; representative development data; risk controls; technical documentation; update and cybersecurity management; post-market surveillance	Model and dataset documentation; validation results; version history; known limitations; incident and corrective-action records
Healthcare institution	Procurement; local validation; workflow integration; staffing; access control; training; monitoring; incident response; vendor governance	Approval decision; local performance and equity results; training and credentialing; contracts; change logs; surveillance dashboards; incident investigations
Clinician and care team	Confirm clinical fit; interpret output in context; communicate material AI use; preserve patient autonomy; override when necessary; report concerns	Clinically relevant inputs and outputs; reasoning when AI materially affects care; meaningful overrides; patient communication; safety reports
Data protection/legal/quality functions	Lawful basis; DPIA; data minimization; consent and transparency materials; regulatory classification; retention; audit and claims readiness	DPIA and records of processing; policies; consent materials; conformity and vendor records; audit findings
Regulators and professional bodies	Minimum safety and evidence standards; surveillance; specialty guidance; education and enforcement	Guidelines; standards; safety communications; registries; enforcement and post-market data

**Table 5:** Actionable institutional requirements for the deployment of AI in gynecology and obstetrics.

Component	Minimum Requirement	Accountable Owner	Verifiable Indicator
1. Responsibility allocation	Register of all clinical AI tools with a named clinical and technical owner; contracts specifying update notification, log access, incident cooperation, and liability allocation	AI governance committee; legal office	All tools registered with named owners; contract checklist completed before go-live
2. Minimum validation	Before routine use, local retrospective validation on representative data (discrimination, calibration, subgroup performance), then a supervised pilot in shadow mode with predefined acceptance and rollback thresholds	Clinical owner; clinical engineering or data science	Validation report approved by the committee; thresholds documented
3. Staff training	Role-specific training before access (intended use, limitations, uncertainty, override, documentation, incident reporting), refreshed after material updates (AI Act, Art. 4)	Training office; department heads	Proportion of users trained before access; competence assessment
4. Documentation and audit	Logs retained for at least six months (AI Act, Art. 26) or longer where medical-record rules require; documentation of material AI use in the clinical record; periodic audit of overrides and alert burden	Clinical risk management; IT department	Audit performed at predefined intervals (e.g., every 6–12 months) with documented actions
5. Equity monitoring	Predefined acceptable performance gaps across age, ethnicity, language, disability, and parity; suspension or restriction of the tool when thresholds are exceeded	AI governance committee; quality office	Subgroup sensitivity, specificity, and calibration reported at each audit
6. Post-deployment surveillance	Monitoring of performance drift, near misses, and adverse events; re-validation after each material update or change in population or equipment	Clinical owner; manufacturer	Drift indicators reviewed at predefined intervals; re-validation records
7. Incident reporting	Non-punitive reporting route for staff and patients; root-cause analysis including technical logs; serious incidents notified to the manufacturer and competent authorities (MDR, Art. 87; AI Act, Art. 73)	Clinical risk management; manufacturer	Time from incident to analysis; corrective actions closed

AI, artificial intelligence; IT, information technology; MDR, Medical Device Regulation.

## Data Availability

No new data were created or analyzed in this study. Data sharing is not applicable to this article.

## Abbreviations

The following abbreviations are used in this manuscript:

AI	Artificial intelligence
EU	European Union
GDPR	General Data Protection Regulation
CTG	Cardiotocography
LLM	Large language model
DPIA	Data protection impact assessment
DPO	Data protection officer
EHDS	European Health Data Space
MDR	Medical Device Regulation
PRISMA	Preferred Reporting Items for Systematic Reviews and Meta-Analyses
SANRA	Scale for the Assessment of Narrative Review Articles
